# Horseflesh for Our Food

**Published:** 1895-02

**Authors:** Rush Shippen Huidekoper


THE JOURNAL

OF

COMPARATIVE MEDICINE AND

VETERINARY ARCHIVES.

VOL. XVI. *FEBRUARY*, 1895. NO. 2.

### HORSEFLESH FOR OUR FOOD.

BY RUSH SHIPPEN HUIDEKOPER, M.D.

HORSEFLESH as an article of food for man is so limited among the English-speaking nations that its very proposal seems to raise a sentiment of repugnance in a great many people which it is difficult to account for.

This feeling appears to be based upon the insular English prejudice against anything which is new or has not been the custom, a characteristic prejudice which is hereditary in the Canadian, American, Australian, and Anglo-Indian of Asia. Upon argument, the objector invariably acknowledges that no reason guides his feelings, but that he has a prejudice against horseflesh.

Among the first evidences of early man, in the dolmans of North Germany, the caves of the Troglodytes of Belgium, France, and Great Britain, there are frequent traces of the bones of the horse among the débris of other animals which were used for food, long before there are traces of the remains of cattle, sheep, or pigs.

In the caves of the early quaternary period at Chatres, France; in the Valley of the Arno; at Aurignac; in the Kjokkenmoeddings of Denmark; at Lourdes, in France, and in the great caves in England, mixed with the bones of early man, together with the primitive stone instruments which he used for his early industries, are the bones of gigantic elephants, rhinoceri, great deer, great bear, some of the large cat tribes, and numerous bones of the horse.

These bones are invariably fragments of the long bones, which have been cracked, as they still show, by the stone hatchets, to obtain the marrow, and remnants of skulls, always broken for the extraction of the brain for food, or, perhaps, also for tanning hides, as is done by the Esquimaux of to-day. Throughout later periods in the quaternary down to historical times, the bony remains of the horse are found in connection with all evidences of civilized man, with marks indicating that they have been used for food.

In historical times the first mention of the use of horseflesh as food comes in the form of its interdiction, Leviticus, xi., 1–3, 7:

"And the Lord spake unto Moses and to Aaron, saying unto them,

"Speak unto the children of Israel, saying, These are the beasts which ye shall eat, among all the beasts that are on the earth.

"Whatsoever parteth the hoof, and is clovenfooted, and cheweth the cud, among the beasts that ye shall eat.

"And the swine, though he divide the hoof, and be clovenfooted, yet he cheweth not the cud; he is unclean to you."

It is absurd in the present century for any one to find support in the Mosaic laws for the non-consumption of horseflesh on the ground that the horse is not clovenfooted and cheweth not the cud, when man, woman, and child, with the exception of the most orthodox of Hebrews, eat ham, sausage, and pig in every form, absolutely ignoring the last verse of the quotation, which distinctly forbids it.

We find from the writing of Herodotus and Leo Africanus that the wild horse was hunted in the chase, and considered a great delicacy by the Arabs and nomadic tribes of Asia and North Africa. In the account of the expedition of the Czar Peter I. against Azof, the wild horse, which under the name of "tarpan" roved in numbers between latitude 30 and 50 degrees, was hunted and used for food by the Tartars, Mongolians, and Manchurians.

In the eighth century Pope Gregory III., and his successor, Zacharias I., sent letters to St. Boniface, the German Apostle, forbidding the use of horseflesh as being allied to ancient religious practices, and therefore an obstacle to the propagation of Christianity. Notwithstanding the Papal edicts, we find that horseflesh was extensively used throughout Central Europe in the succeeding centuries and in the middle ages, and at times formed an important article of diet, but at times only, as domesticated cattle were then obtainable in larger numbers, and the horse has assumed greater value for war, for the chase, and for other sports.

Following the middle ages, for the same reason, the use of horseflesh for food was more limited, and was only practised in restricted localities and in time of war or famine. About the middle of the present century its use was made the subject of governmental investigation and control, as a matter of health, following the laws which had been but recently formulated regulating the inspection and supervision of the sale of beef.

In 1866 laws were passed by the French Government ordering that horses should only be killed in special slaughter-houses; that they should only be killed in the presence of a veterinarian or authorized inspector, who should verify the healthy condition of all of the viscera; that the meat should only be sold after being officially stamped, and that the meat should only be sold as horsemeat in shops which bore over the door a sign in large letters to that effect; the peddling of horseflesh was forbidden.

The first authorized shop for the sale of horsemeat was opened in Paris, July 9, 1866. In the remaining six months of that year there were sold for food 902 horses; in 1867, 2069 horses, 59 asses, and 24 mules; in 1868, 2297 horses, 97 asses, and 11 mules. In 1870 and 1871, during the period of the siege of Paris and the Commune, the consumption increased to 64,362 horses, 635 asses, and 3 mules, a total of 65,000, or about 24,000,000 pounds of dressed meat, averaging 384 pounds per horse.

In 1872 there were consumed 5034 horses, 675 asses, and 23 mules; in 1875, 6448 horses, 394 asses, and 23 mules; in 1878, 10,800 horses, 488 asses, and 31 mules, and since that time there has been a steady increase not only in Paris, but also in the other large towns of France. Since 1870 there has been an equally constant increase in the use of horsemeat for food in Brussels, Antwerp, Cologne, Berlin, and the larger German cities.

In 1882, upon the failure of one of the largest restaurants in

Berlin, in which the best of marketing was supposed to be sold, it was found that one of the largest creditors was a dealer in horsemeat, who had been supplying the restaurant for years with meat, which the restaurant served as beef.

The nutritive value of the meat of the horse is given by Leyder and Pyro, for a horse in moderate condition as follows:

| | Neck. | Filet. | Thigh. |
|---|---|---|---|
| Water | 75.02 | 76.00 | 75.22 |
| Fixed matter | 24.08 | 24.00 | 24.08 |
| Muscular matter | 22.85 | 21.76 | 23.26 |
| Fat | 0.95 | 1.24 | 0.52 |
| Ash | 1.00 | 1.00 | 1.00 |

In comparing this with the nutritive qualities of the flesh of beef in good condition, and of "piners" or "Bologna" cattle, it will be found that the fixed matter is poorer than in good beef, but richer than in poor beef. The amount of fat is approximately the same in a horse and an ox in the same condition.

Liebig found in 100 kilogrammes of horsemeat 72 grammes of creatine, and in 100 kilogrammes of beef 62 grammes of creatine, and he claimed for the horseflesh the greater nutritive value. A horse in fair condition, weighing 1000 pounds on the hoof, dressed 547½ pounds of meat with the larger bones, giving about the same percentage as that of fair beef.

The following is a comparison of the physical characters of the properly dressed meat of the horse and ox:

| Ox | Horse. |
|---|---|
| Color—Bright red. | Color—Dark red. |
| Consistence—Firm, becoming soft and unctuous later. | Consistence—Firm and hard. |
| On Cutting—Easy, fine grain. | On Cutting—More resisting, coarser grain. |
| Odor—Fresh, somewhat aromatic | Odor—Slightly musky. |
| Fat—White or yellowish. | Fat—yellowish. |

In reality the flesh of the horse differs but little from that of beef of equal quality. It has nothing disagreeable to the eye or to the taste. It makes a consomme rather less clear and bright than that of beef, and the meat loses rather more color in boiling; but after broiling or roasting, in which way horsemeat should always be cooked, it has no appearance by which it can be detected from beef. There is, perhaps, a slightly sweetish taste, which, however, is entirely overcome by the salt, pepper, and sauces which are usually eaten with roast meat,

The flesh of the ass and mule has a finer grain than that of the horse, and has a very slight "gamy" taste, which, however, is scarcely to be distinguished from a prime rump steak.

The celebrated "Bologna" sausage, when properly made, as it originally was in Italy, is made from donkey's meat only. The majority of that in the market to-day is made from the poorest grades of beef, mixed with other cheap meats, pork, etc.

Baron Larrey, the celebrated surgeon of Napoleon I., was an enthusiast on the merits of horseflesh, and his belief in its advantages was probably strengthened by the service which it rendered in feeding the troops of Napoleon's armies. The great savant, Geoffrey St. Hilaire, was also an ardent advocate of the use of horsemeat for reasons of utility and economy.

Numerous dinners have been given at various times by individuals and hippophagic societies, where horsemeat has been served alone, cooked in various forms, or side by side with beef, each course having both meats cooked in the same manner. Undoubtedly the meats are so similar that in the majority of cases they are not to be distinguished, especially if slightly disguised by the sauces and dressings which the cook's art has so completely perfected. Enthusiastic hippophagists frequently go so far as to claim that the horsemeat is preferable.

Some years ago I owned a rather celebrated steeplechaser which had been at pasture for several years, but which had a chronic trouble of the feet, which was incurable and caused her so much pain that it was a charity to kill her. Her meat looked so fresh and tempting that I had her fillets removed and sent to my club, with instructions to the steward that we would have them for dinner.

A friend was instructed to call a meeting of a dozen men to dine and discuss some questions then under consideration. The fillet was served a la Bordelaise. A recent change of the cooks in the club served readily as an excuse for criticism of the cooking and for calling attention to the fillet, which was pronounced to be perfect. Some time after the coffee the guests were told that the fillet was from the old steeplechaser, whom they had all seen win many silver cups. The effect on my friends was amusing. One of the guests, who had been most ardent in praise of the new cook, found immediate fault, and continued to for some days. This certainly was prejudice.

I have had a great deal of experience myself in the use of the flesh of the horse, ass, and mule, and, while I do not agree that it is as good as good beef, I think there are many reasons why horsemeat should become an article of diet, and be regularly and properly supplied in markets legalized for its sale.

I do not consider its taste equal to that of good beef, but I do consider it superior to poor beef. In its nutritive qualities it is equal to beef when the "condition" of the animals is equal. It is assumed that the horse to be used is in perfectly healthy condition, free from any fever, disease of the lungs or other viscera, and has no local inflammation or abscess Chronic local troubles of the bones or tendons of the legs, simply causing lameness, need not be considered as disease.

The horse has no constitutional disease which is communicable to man, except glanders, which can always be absolutely diagnosed by post-mortem inspection. Cattle, on the other hand, are frequently infected with tuberculosis, which cannot always be detected by microscopic examination or by the ordinary inspections in the slaughter-house. Gray horses are usually refused at the markets for horses for food, as they are apt to be affected by melanotic tumors, which, while not unhealthy, render the meat gritty and disagreeable.

The value of a horse in prime condition is usually too great to make it a competitor of prime beef, but numbers of horses, because of age, or local troubles causing lameness, fall to a value where they can be readily utilized to great advantage in competition with the poorer qualities of beef. The meat from these horses can be profitably sold cheaper than beef, it is equally nutritious, and brings within the reach of the poorer classes a healthy, good animal food, which they would do without or have less frequently if obliged to pay the price of beef.

From a humanitarian point of view, the opening of markets for horses would give an outlet to many which were just commencing their downward course in value, and it would also be a happier ending for them than the street express wagon and huckster's cart, to which the falling animal rapidly comes to end a useful life with poor food and under cruel blows.

---

THE veterinary department of the University of Pennsylvania has a corps of nineteen instructors for the session of 1894–'95, an increase of three over the preceding year. The entire number of students reaches seventy-eight, and was the same for the preceding year.

